# Preclinical to Clinical Translation of CNS Transporter Occupancy of TD-9855, a Novel Norepinephrine and Serotonin Reuptake Inhibitor

**DOI:** 10.1093/ijnp/pyu027

**Published:** 2015-01-29

**Authors:** Jacqueline AM Smith, DL Patil, OT Daniels, Y-S Ding, J-D Gallezot, S Henry, KHS Kim, S Kshirsagar, WJ Martin, GP Obedencio, E Stangeland, PR Tsuruda, W Williams, RE Carson, ST Patil

**Affiliations:** Theravance Biopharma US, Inc., San Francisco, CA (Drs Smith, Bourdet, Daniels, Kim, Kshirsagar, Martin, Obedencio, Stangeland, Tsururda, Williams, and Patil); Yale School of Medicine, New Haven, CT (Drs Ding, Gallezot, Henry, Williams, and Carson).

**Keywords:** norepinephrine and serotonin transporter, pain, PET, TD-9855

## Abstract

**Background:**

Monoamine reuptake inhibitors exhibit unique clinical profiles that reflect distinct engagement of the central nervous system (CNS) transporters.

**Methods:**

We used a translational strategy, including rodent pharmacokinetic/pharmacodynamic modeling and positron emission tomography (PET) imaging in humans, to establish the transporter profile of TD-9855, a novel norepinephrine and serotonin reuptake inhibitor.

**Results:**

TD-9855 was a potent inhibitor of norepinephrine (NE) and serotonin 5-HT uptake *in vitro* with an inhibitory selectivity of 4- to 10-fold for NE at human and rat transporters. TD-9855 engaged norepinephrine transporters (NET) and serotonin transporters (SERT) in rat spinal cord, with a plasma EC_50_ of 11.7ng/mL and 50.8ng/mL, respectively, consistent with modest selectivity for NET *in vivo*.

Accounting for species differences in protein binding, the projected human NET and SERT plasma EC_50_ values were 5.5ng/mL and 23.9ng/mL, respectively. A single-dose, open-label PET study (4–20mg TD-9855, oral) was conducted in eight healthy males using the radiotracers [^11^C]-3-amino-4- [2-[(di(methyl)amino)methyl]phenyl]sulfanylbenzonitrile for SERT and [^11^C]-(*S,*S)-methylreboxetine for NET. The long pharmacokinetic half-life (30–40h) of TD-9855 allowed for sequential assessment of SERT and NET occupancy in the same subject. The plasma EC_50_ for NET was estimated to be 1.21ng/mL, and at doses of greater than 4mg the projected steady-state NET occupancy is high (>75%). After a single oral dose of 20mg, SERT occupancy was 25 (±8)% at a plasma level of 6.35ng/mL.

**Conclusions:**

These data establish the CNS penetration and transporter profile of TD-9855 and inform the selection of potential doses for future clinical evaluation.

## Introduction

Dose selection for central nervous system (CNS)-based therapeutics is guided by many factors, including target engagement and the underlying pathophysiology of the disease of interest. For monoamine reuptake inhibitors, where unique clinical profiles emerge from distinct CNS monoamine transporter engagement, the challenge of optimal dose selection is amplified. TD-9855 is an investigational dual norepinephrine (NE) and serotonin (5-HT) reuptake inhibitor with selectivity for NE over 5-HT (NSRI). Dual monoamine reuptake inhibitors offer an opportunity to relieve core symptoms and associated comorbidities across a spectrum of CNS disorders by differential regulation of NE and 5-HT. Here, we used a translational strategy that included rodent pharmacokinetic/pharmacodynamic (PK/PD) modeling and positron emission tomography (PET) imaging in humans to establish the profile of TD-9855. The systematic integration of *in vitro* and *in vivo* preclinical data with clinical PET imaging data can provide a quantitative assessment of the CNS transporter profile for monoamine reuptake inhibitors. Since differential transporter engagement may confer therapeutic benefit in different patient populations, our objectives were to confirm central penetration and to inform selection of potential doses of TD-9855 for clinical evaluation.

Central NE levels influence a wide range of neurobiological functions, including mood, attention, and pain (Millan, 2002; Berridge and Waterhouse, 2003; Robbins and Arnsten, 2009; Del et al., 2011; Hamon and Blier, 2013). The multiple actions of NE underscore the diverse therapeutic benefits of NE reuptake inhibitors, which are approved to manage conditions such as major depressive disorder (MDD; Schatzberg, 2000) and attention deficit hyperactivity disorder (ADHD; Spencer et al., 2002; Michelson et al., 2003), respectively. While MDD and ADHD are distinct neuropsychological disorders, common features such as cognitive impairment may share the same mechanistic underpinning (Chamberlain and Robbins, 2013). Indeed, deficits in attention and executive functioning may be important features of many conditions, suggesting that reuptake inhibitors with noradrenergic activity may offer broader therapeutic benefit (Glass, 2009; Vazey and Aston-Jones, 2012; Adler, 2013; Marsh et al., 2013).

By its very nature, pain represents a multidimensional experience in which emotional and cognitive dimensions converge with sensory elements to signal actual or potential tissue damage (Neugebauer et al., 2009). Norepinephrine and 5-HT are major components of the descending pain inhibitory control system that runs from the brain stem to the spinal cord (Basbaum and Fields, 1984). In the setting of chronic pain, central modulatory pathways may become dysregulated, yielding altered or reduced levels of NE and 5-HT at supraspinal and/or spinal levels. Dual 5-HT and NE reuptake inhibitors, such as duloxetine (Cymbalta®), are approved for the management of chronic pain conditions as well as for conditions that can be co-morbid with pain, like anxiety and depression. Duloxetine has significant serotonergic activity, exhibiting at least 10-fold selectivity *in vitro* for inhibition of serotonin (SERT) over norepinephrine transporters (NET). At the 60mg dose, duloxetine exhibits >80% occupancy of SERT (Takano et al., 2006). Milnacipran (Savella®) is a dual reuptake inhibitor with more apparent noradrenergic activity than duloxetine, but likely insufficient selectivity between NET and SERT to enable differential engagement of each transporter (Tsuruda et al., 2010; Nogami et al., 2013). NE reuptake inhibitors exhibit efficacy in chronic pain but, in contrast, selective 5-HT reuptake inhibitors (SSRIs) do not produce a primary analgesic effect (Max et al., 1992; Fishbain et al., 2000; Finnerup et al., 2005; Atkinson et al., 2007; Verdu et al., 2008; Arnold et al., 2012). SSRIs can augment the activity of a selective NE reuptake inhibitor (Fishbain et al., 2000; Iyengar et al., 2004; Finnerup et al., 2005; Hall et al., 2011), and we hypothesize that an inhibitor with modest selectivity for NET instead would offer the potential for robust pain relief while minimizing any putative serotonergic side effects such as nausea, somnolence, fatigue, and sexual dysfunction (Papakostas, 2008).

The objective of the present study was to build a translational understanding of the preclinical and clinical PK/PD properties of TD-9855 that would establish its CNS transporter profile. The *in vitro* pharmacology profile of TD-9855 was determined at both rat and human monoamine transporters. Rat CNS *ex vivo* occupancy studies were used to characterize the kinetics and dynamics of changing TD-9855 concentrations in the plasma and CNS, and enable construction of a PK/PD model describing the relationship between TD-9855 plasma concentration and transporter occupancy (Grimwood and Hartig, 2009; Bourdet et al., 2012). Central penetration, relative levels of SERT and NET occupancy, and the relationship between plasma concentration and occupancy were evaluated in a Phase 1, open-label, single-dose PET study in healthy volunteers using the radiotracers [^11^C]-3-amino-4-[2-[(di(methyl)amino)methyl]phenyl]sulfanylbenzonitrile ([^11^C]-DASB; for SERT) and [^11^C]-(*S,*S)-methylreboxetine ([^11^C]-MRB; for NET). Development of a NET radioligand suitable for PET imaging has been limited and proven challenging (Ding et al., 2006). The most promising chemical structures are in the MRB class, and [^11^C]-MRB has emerged as a radioligand that enables some quantitation of NET occupancy in the human brain (Hannestad et al., 2010; Pietrzak et al., 2013; Li et al., 2014). The long human PK half-life (30–40h) of TD-9855 (Theravance Biopharma US, Inc., data on file) provided a unique opportunity for same-subject sequential assessment of SERT and NET occupancy. Here we summarize the findings from translational development of TD-9855 from the preclinical to Phase 1 stages and report doses of TD-9855 that yield single-target engagement of NET only or dual engagement of NET and SERT to support dose selection for a Phase 2 proof-of-concept study.

## Methods

### Animals

Adult male Sprague Dawley rats (Charles River) were housed under controlled laboratory conditions (temperature at 21±1ºC) on a 12:12 hour light-dark cycle. Animals were given free access to food and water upon arrival to the facility and animals were acclimatized to their holding room for at least 48 hours. Animals were fasted but allowed free access to water for 15–18 hours prior to dosing. All animal experiments were approved by the Institutional Animal Care and Use Committee at Theravance Biopharma US, Inc.

### Equilibrium Radioligand Binding and [^3^H]-Neurotransmitter Uptake

**Figure 1.**
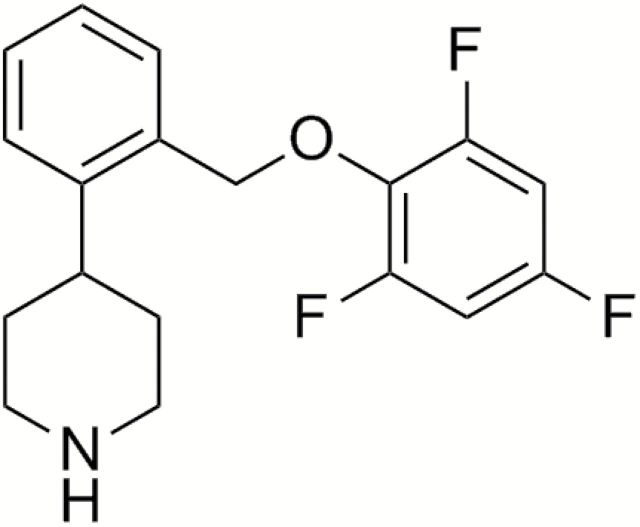
TD-9855, 4-[2-(2, 4, 6-trifluorophenoxymethyl)phenyl]piperidine.

The *in vitro* pharmacology of TD-9855 (4-[2-(2, 4, 6-trifluorophenoxymethyl)phenyl]piperidine; Figure 1) at human recombinant and rat native monoamine transporters was characterized as described previously (Tsuruda et al., 2010; Shen et al., 2013). Radioligands were sourced commercially (Perkin Elmer Life Sciences or GE Healthcare Life Sciences). Briefly, membranes prepared from HEK293 (Human Embryonic Kidney 293) cells stably-transfected with human recombinant SERT (HEK293-hSERT), NET (HEK293-hNET), or DAT (CHO-K1-hDAT) were incubated for 1hr at 22°C in the absence, or presence, of TD-9855 and [^3^H]-citalopram (1.0nM) for SERT, [^3^H]-nisoxetine (2.0nM) for NET, and [^3^H]-WIN35428 (3.0nM) for DAT in 50mM Tris-HCl, 120mM NaCl, 5mM KCl, 0.025% BSA, 100 μM ascorbic acid, pH 7.4. Rat cortical membrane preparations were incubated with [^3^H]-citalopram (2.0nM) for SERT or [^3^H]-nisoxetine (4.0nM) for NET for 1hr at 22°C. In neurotransmitter uptake assays HEK293-hSERT, hNET, or hDAT cells, respectively, were pre-incubated for 30min at 37°C in the absence, or presence, of TD-9855 in 7.5mM HEPES, 12.5mM Tris–HCl, 2.2mM Na-phosphate, 120mM NaCl, 5mM KCl, 0.4mM MgCl_2_, 7.5mM glucose, 1.7mM CaCl_2_, 250 μM ascorbic acid, 150 μM pargyline, 0.025% BSA, pH 7.4 prior to incubation with [^3^H]-5-HT (20nM), [^3^H]-NE (40nM), or [^3^H]-DA (100nM) for 10min. Rat cortical synaptosomes were incubated with [^3^H]-5-HT or [^3^H]-NE for 6min and striatal synaptosomes with [^3^H]-DA for 6min. Binding and uptake assays were terminated by rapid filtration and radioactivity, determined by liquid scintillation spectroscopy.

Final [^3^H]-neurotransmitter concentrations were significantly below the respective K_m_ such that pIC_50_ approximated functional pK_i_. Selectivity for NET (rounded to one significant figure) was determined as follows: selectivity = 10^(pKi or pIC50 at NET minus pKi or pIC50 at SERT or DAT)^.

**TD-9855 Rat Administration and Sampling** *—* Rats (n = 6/timepoint/dose level) received a single oral dose of TD-9855 (0.3, 1, 5, 10, 30, and 60mg/kg) and were euthanized by decapitation at specified time points (0.5, 2, 4, 6, and 8hr for 5mg/kg dose level; 2hr for 0.3, 1, 10, 30, and 60mg/kg dose levels) post-administration. Spinal cords were dissected for *ex vivo* transporter occupancy and PK assessments from the same animals. The spinal cord was harvested by hydraulic extrusion using phosphate-buffered saline, and the lumbar segment dissected and frozen on dry ice. The remaining spinal cord segments were collected and homogenized in water (25% w/w) for PK analysis. All samples were stored at -80ºC until analysis.

### *Ex Vivo* Transporter Occupancy

A kinetic radioligand binding assay was used to determine NET and SERT occupancy in rat spinal cord, as described previously (Bourdet et al., 2012).

**Rat PK/PD Modeling** — PK/PD parameters were estimated by a compartmental modeling approach (WinNonlin Version 5.0.1, Pharsight Corporation). One- and two-compartment PK models with first-order absorption and elimination were evaluated. The one-compartment model was selected. The pharmacodynamic model was an effect compartment E_max_ model linked directly to the central PK compartment (WinNonlin PK Model 3, PD Model 101). Selection of models was based upon best fit in terms of visual inspection, Akaike Information Criteria, and weighted residual sum of squares using the Gauss-Newton minimization method. The following parameters were estimated:

k01 (hr^-1^): First-order absorption rate constant.

V/F (L/kg): Volume of the central compartment divided by oral bioavailability

k10 (hr^-1^): Elimination rate constant from the central compartment

E_max_ (% occupancy): Maximal SERT or NET occupancy in spinal cord

EC_50_ (ng/mL): Plasma TD-9855 concentration associated with 50% SERT or NET occupancy

k_eo_ (hr^-1^): First-order equilibration rate constant between the central pharmacokinetic compartment and the pharmacodynamic effect compartment

### Phase 1: Human PET Study

This was a Phase 1, open-label, single-dose study in healthy male volunteers to evaluate NET and SERT occupancy in the human brain after oral administration of TD-9855 using PET.

The study was conducted at the Yale PET Center in accordance with the principles of the International Conference on Harmonisation’s Technical Requirements for Registration of Pharmaceuticals for Human Use Guideline for Good Clinical Practice. The protocol was approved by the Yale University Human Investigation Committee and the Yale–New Haven Hospital Radiation Safety Committee. SERT and NET occupancy were determined using the selective radiotracers [^11^C]-DASB (Meyer et al., 2004; Takano et al., 2006; Abanades et al., 2011; Nogami et al., 2013) and [^11^C]-MRB. Selection of the radiotracers and the dose of TD-9855 were flexible to minimize the number of PET scans.

Eligible subjects were enrolled and received either [^11^C]-MRB and/or [^11^C]-DASB. Each subject underwent up to 5 PET scans over a period of up to 8 days. Multiple dose levels of TD-9855 (up to 20mg) were explored. All randomized subjects received the NET tracer (single-tracer evaluation) and some subjects (n = 3) received both the NET and SERT tracers sequentially (dual-tracer evaluation) in the baseline and post-dose period. For single-tracer evaluation, subjects completed the first PET scan at baseline, a second PET scan on Day 1 from 7–10hr (corresponding to Tmax; range 6 to 12 hours; in the ascending single dose studies; Theravance Biopharma US, Inc., data on file) following dosing of TD-9855, and a follow-up PET scan on or between Days 3 and 7. In the dual-tracer evaluation, subjects completed sequential [^11^C]-DASB and [^11^C]-MRB baseline scans with each tracer, sequential post-dose scans with each tracer at 7–10hr post-dose, and one follow-up scan with [^11^C]-MRB on or between Days 3 and 7.

Each subject received one dose of TD-9855, a maximum of 75 millicuries (mCi) of radioactivity, and five injections.

Only healthy male subjects (age-range of 18–45) were eligible to participate. Key exclusion criteria included no prior exposure to monoamine reuptake inhibitors or stimulants or a history of smoking. Subjects with magnetic resonance imaging (MRI)-incompatible implants and other contraindications for MRI were also excluded.

All adverse events (AEs) were assessed by the investigator and recorded in the case report form, including the dates of onset and resolution, severity, relationship to study drug, outcome, and action taken with study medication after the dosing of TD-9855 through the follow-up visit. Safety was assessed during the entire study by measurement of vital signs, laboratory tests, and evaluation of ECGs.

### PET Imaging and Parameters

The injected dose of both tracers was typically 14–15 mCi, with mass doses less than 10 µg in all cases. PET images were acquired using the High Resolution Research Tomograph (HRRT; Siemens/CTI) with a reconstructed image resolution of approximately 3mm. Following a transmission scan, [^11^C]-MRB or [^11^C]-DASB was injected intravenously as a 1min bolus by an infusion pump. List-mode data were acquired for 120min. Head motion correction was performed using an optical tracking tool (Vicra, NDI Systems) and a rigid tool attached to a swim cap.

Dynamic scan data were reconstructed with corrections for attenuation, normalization, scatter, randoms, dead time, and motion using the MOLAR algorithm (motion-compensation ordered subset-expectation maximization list-mode algorithm for resolution recovery reconstruction) with the following frame timing: 6 x 30sec; 3 x 1min; 2 x 2min; 22 x 5min.

### Plasma PK Measurements

Four 6mL samples were drawn at 2, 4, 6, and 24hr post TD-9855 dosing. Three additional 6mL samples were drawn prior to each PET scan and approximately 60 and 120min after the beginning of each PET scan. Plasma samples were analyzed for TD-9855 concentrations by a validated LC-MS/MS method. The lower limit of quantification for TD-9855 was 0.05ng/mL.

### Image Analysis

The occipital cortex was selected as the reference region for [^11^C]-MRB due to the low density of NET measured *in vitro* (Schou et al., 2005) and the absence of specific binding in a previous study (Hannestad et al., 2010). The regions of interest (ROIs) selected to assess [^11^C]-MRB specific binding included one cortical region and the paracentral lobule, thalamus, hypothalamus, locus ceruleus, nucleus rubra, and raphe nucleus. The thalamus and cortical ROIs were delineated using the Automated Anatomical Labeling (AAL) template (Tzourio-Mazoyer et al., 2002). The hypothalamus, locus ceruleus, nucleus rubra, and raphe nucleus ROIs were drawn on the template MR image, based on the AAL template (Hannestad et al., 2010).

For [^11^C]-DASB, the cerebellum was used as the reference region. The regions of interest selected to assess [^11^C]-DASB specific binding were the caudate, putamen, amygdala, and thalamus. All regions were delineated using the AAL template (Tzourio-Mazoyer et al., 2002).

### MR and PET Image Processing

In order to apply the ROIs defined on the template MR image to the PET images to compute regional time-activity curves (TACs), two geometric transforms were estimated:

1. One nonlinear deformation field was estimated between the template MR image and each subject’s MR image (3D MPRAGE) to account for inter-subject anatomical variability. The nonlinear deformation was estimated using the BioImage Suite software Version 2.5 (http://www.bioimagesuite.org/).
2. One rigid linear coregistration matrix was estimated between each subject’s MR image and each PET scan to account for subject positioning. The linear transformation was estimated using the FLIRT (FMRIB’s Linear Image Registration Tool) software (http://www.fmrib.ox.ac.uk/analysis/research/flirt/).

### Computation of Binding Potentials

For both studies, binding potentials (*BP*_ND_) were estimated using a method based on a reference region input curve. For [^11^C]-MRB regional *BP*_ND_ were computed using the multilinear reference tissue model 2 (MRTM2; Ichise et al., 2003). The suitability of MRTM2 to estimate [^11^C]-MRB *BP*_ND_ values was evaluated in a previous study (Hannestad et al., 2010), in which it was shown that MRTM2 *BP*_ND_ estimates are highly correlated with *BP*_ND_ estimated with arterial blood sampling and the multilinear analysis 1 (y = 0.909 x + 0.078, r^2^ = 0.821, where x represents the MA1 *BP*_ND_ values and y represents the MRTM2 *BP*_ND_ values). *BP*_ND_ values and their associated standard errors ( $SE_{BP_{ND}}$ ) for each ROI were then estimated by performing fits of the regional TACs. Data were fitted starting at time t* = 20min. For [^11^C]-DASB, regional *BP*_ND_ values were computed using simplified reference tissue model 2 (SRTM2; Wu and Carson, 2002). SRTM2 is similar to MRTM2, except that it uses the full data set, while MRTM2 uses the period of time t > t*. When applied to single-voxel TACs, these fits provided the *BP*_ND_ images. Before computing parametric images, the original dynamic images were smoothed with a Gaussian filter with a full width at half maximum of 3 voxels (3.7mm).

### Computation of Apparent Occupancy

Apparent occupancy values ( $r_{\text{App}}^{\text{ROI}}$ ) for individual ROIs and their associated standard errors ( $SE_{r_{\text{App}}^{\text{ROI}}}$ ) were computed for each PET scan after administration of TD-9855 as follows:

$$r_{App}(\%)=100*\left[1-\frac{BP_{ND}^{\text{Post drug}}}{BP_{ND}^{\text{Baseline}}}\right]$$

and

$$SE_{r_{\text{App}}^{\text{ROI}}}=100\times \sqrt{{\left(\frac{SE_{BP_{ND}^{Postdrug}}}{BP_{ND}^{Baseline}}\right)}^{2}+{\left(\frac{SE_{BP_{ND}^{Baseline}}\times BP_{ND}^{Postdrug}}{{\left(BP_{ND}^{Baseline}\right)}^{2}}\right)}^{2}}$$

A whole-brain apparent occupancy ( $r_{\text{App}}^{}$ ) was computed for each post-drug scan as the weighted average of the regional apparent occupancies:

$$r_{App}=\sum_{ROIs}r_{App}^{ROI}/SE_{r_{App}^{ROI}}^{2}/\sum_{ROIs}1/SE_{r_{App}^{ROI}}^{2}$$

The $SE_{r_{\text{App}}^{}}^{}$ of the $r_{\text{App}}^{}$ was computed using the error propagation equation (Bevington and Robinson, 2014) as follows:

$$SE_{r_{\text{App}}^{}}=\sqrt{\frac{1}{\sum \frac{1}{SE_{r_{\text{App}}^{\text{ROI}}}^{2}}}}$$

The EC_50_ of TD-9855 for NET was estimated by fitting the *r*_App_ values for [^11^C]-MRB, with weights equal to $1/SE_{r_{\text{App}}^{}}^{2}$ , versus the average plasma concentration of TD-9855 during the scan (*C*) with a two-parameter model, including the drug EC_50_ and the tracer maximal apparent occupancy (*r*_max_):

$$r_{App}(\%)=r_{\max}\left(\frac{C_{\;}}{C_{\;}+EC_{50}}\right)$$

Note that either simultaneously fitting all the regional occupancy estimates $r_{\text{App}}^{\text{ROI}}$ with weights equal to $1/SE_{r_{\text{App}}^{\text{ROI}}}^{2}$ or fitting the *r*_App_ estimates with weights equal to $1/SE_{r_{\text{App}}^{}}^{2}$ leads to the same EC_50_ and *r*_max_ estimates up to a constant offset, since the cost functions are identical.

Theoretical maximal occupancy should be 100%; however, to account for the effects of small regional differences in nonspecific binding, the variable *r*_max_ was included. Using the estimate of the [^11^C]-MRB *r*_max_, the final normalized occupancy estimates for each scan were obtained as r=rApprmax . The maximal apparent occupancy (*r*_Ap__p_) would be 100% if the non-displaceable binding were the same in the reference and target tissues. However, it would be greater than 100% if the non-displaceable binding were lower in the target region, and less than 100% if the non-displaceable binding were higher in the target region, relative to the reference region. This sensitivity of *r*_App_ to differences in non-displaceable binding is more significant for tracers with low specific binding, such as [^11^C]-MRB.

The TD-9855 estimated EC_50_ value and the associated standard error obtained from the fit of the occupancy estimates as a function of the plasma concentration were determined.

## Transporter Occupancy Projections after Repeated TD-9855 Administration

NET occupancy was simulated after repeated TD-9855 administration using a population PK model based upon the PK of TD-9855 observed in single and multiple dose clinical trials (Theravance, data on file). Steady-state TD-9855 plasma concentration versus time profiles were simulated for dose levels of 4mg and 20mg and used in conjunction with the NET occupancy versus plasma concentration model estimates (using *r*_max_ = 100% and EC_50_ = 1.21ng/mL) to derive the mean (95% CI) NET occupancy at steady state.

## Results

### *In Vitro* Pharmacological Profile of TD-9855

The *in vitro* pharmacological profile of TD-9855 was similar at human and rodent monoamine transporters SERT (Table 1). Radiolabelled neurotransmitter uptake studies using cell lines expressing human NET, SERT, and DAT demonstrated that TD-9855 is a potent inhibitor of NET and SERT, but not DAT, with 4-fold higher potency for inhibition of NET over SERT. Similarly, TD-9855 is a potent inhibitor of both [^3^H]-NE and [^3^H]-5-HT uptake into rat cortical synaptosomes, with an apparent functional selectivity (10-fold) for NET over SERT, similar to that observed at human transporters. Consistent with the functional inhibition studies, TD-9855 exhibited a high affinity for binding to human NET and SERT, but not DAT (Table 1). Apparent binding affinity values for rat-native NET and SERT in membranes prepared from rat cortices were similar (overlapping confidence intervals) to the corresponding values at human transporters, consistent with a lack of species dependence (Table 1).

**Table 1.** *In Vitro* Pharmacology of TD-9855 at Human Recombinant and Rat Native SERT, NET, and DAT Transporters.

Species	Neurotransmitter Uptake Inhibition: pIC_50_			Transporter Binding: pK_i_		
	SERT	NET	DAT	SERT	NET	DAT
Human	8.0 (7.8, 8.2)	8.6 (8.4, 8.7)	6.8 (6.6, 6.9)	8.5 (8.5, 8.6)	8.8 (8.8, 8.9)	6.7 (6.7, 6.8)
Rat	7.9 (7.8, 7.9)	8.9 (8.6, 9.1)	6.9 (6.8, 6.9)	8.5 (8.3, 8.6)	8.7 (8.5, 8.9)	N.D.

Data are expressed as mean pIC_50_ (negative decadic logarithm IC_50_) and pK_i_ (negative decadic logarithm K_i_) values. Data represents mean (with 95% confidence intervals in parentheses) from 3 to 9 individual experiments. N.D., not determined.

### Rat PK/PD Modeling

**Figure 2.**
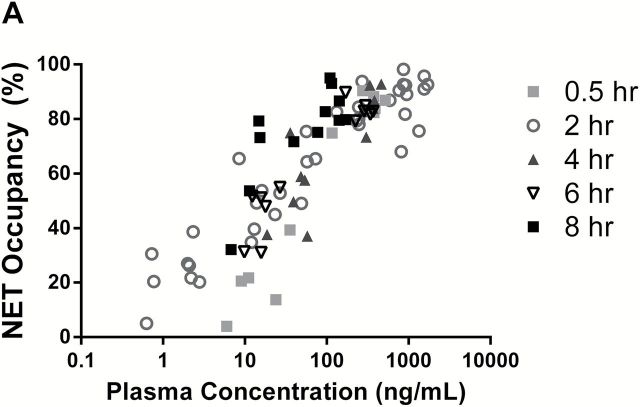

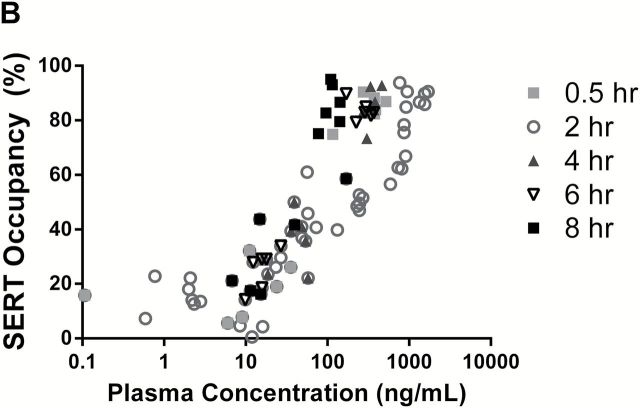
Relationship between TD-9855 plasma concentration and rat spinal cord occupancy at NET (A) and SERT (B). Spinal cord transporter occupancy and TD-9855 plasma concentration were determined at the indicated time-points post-dose (0.3 to 60mg/kg, oral). Symbols represent individual data from all dose levels at the indicated timepoints (n = 4–6/timepoint/dose level).

The relationship between TD-9855 plasma concentration and NET and SERT central occupancy in rats is presented in Figure 2. Data from all dose levels were pooled in this analysis and depicted by the post-dose time-point, at which the occupancy and plasma concentration were measured irrespective of dose level. A PK/PD model of the time course of plasma concentration and NET/SERT occupancy over time was constructed. The model consisted of a one-compartment oral absorption pharmacokinetic model linked directly to an effect compartment sigmoidal E_max_ model. PK and PD parameter estimates derived from the effect compartment PK/PD analysis for NET and SERT occupancy are presented in Table 2. The estimated EC_50_ for occupancy was 11.7ng/mL for NET and 50.8ng/mL for SERT in rat spinal cords. Accounting for species differences in plasma protein binding (90.2% and 79.1% in rat and human, respectively; Theravance Biopharma US, Inc., data on file), the projected human plasma EC_50_ values were 5.5ng/mL for NET and 23.9ng/mL for SERT.

**Table 2.** Pharmacokinetic and Pharmacodynamic Parameter Estimates for TD-9855 Norepinephrine and Serotonin Transporter Occupancy in Rat Spinal Cord.

Parameter	E_max_ (% Occupancy)	EC_50_ (ng/mL)	k_eo_ (hr^-1^)	K01 (hr^-1^)	K10 (hr^-1^)	V/F (L/kg)
SERT	79.0 (53)	50.8 (87)	11.0 (86)	0.777 (108)	0.319 (81)	54.8 (66)
NET	92.0 (19)	11.7 (6.8)	1.78 (57)			

Final parameter estimates are listed with the coefficient of variation (% CV) on each parameter estimate provided in parentheses.

### Phase 1 Human Positron Emission Study

A total of eight subjects completed the study. The study participants were healthy males, aged 29±5 years, and from diverse ethnic and racial backgrounds. Five of the study participants received a single tracer ([^11^C]-MRB) and three of the study participants received two tracers ([^11^C]-MRB and [^11^C]-DASB). Orally administered doses of TD-9855 were 4mg (n = 3), 10mg (n = 1), and 20mg (n = 4). The projected human plasma EC_50_ values from the rat PK/PD modeling were used to select doses that would provide a range of NET occupancy in human CNS.

**Figure 3.**
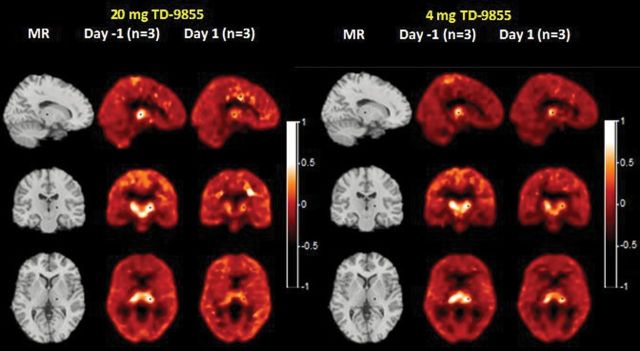
Average PET binding potential (*BP*_ND_) images of NET with [^11^C]-MRB with a single dose of 20mg or 4mg. Columns 1 and 4 show MR images in sagittal, coronal, and transverse orientations. Columns 2, 3, 5 and 6 show [^11^C]-MRB binding potential images at baseline and 9.5 to 11.5 hours after administration of 20mg and 4mg of TD-9855. + shows the location of the orthogonal slices, cut through the thalamus.

**Figure 4.**
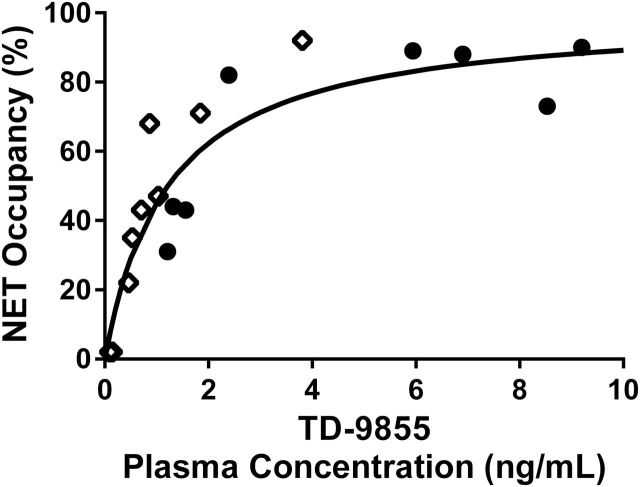
NET estimated occupancy determined for each subject as a function of the drug plasma concentration. The circles represent the data estimated on Day 1; the diamonds represent the data estimated on Days 3 to 7. Each occupancy value represents a weighted average across regions, where the weighting accounts for the uncertainty of the underlying *BP*_ND_ estimates. The apparent occupancy data, listed in Table 3, were fitted to a 2-parameter occupancy model to determine EC_50_ and *r*_max_. Apparent occupancy data then were normalized to a maximum occupancy of 100% (i.e., divided by *r*_max_), as described in Methods, and represented graphically. The solid line represents the best fit, with a global EC_50_ (1.21±0.21ng/mL).

Average NET *BP*_ND_ images at the level of the thalamus are shown at baseline and at 9.5–11.5hr, post-administration of either 4mg or 20mg of TD-9855, respectively (Figure 3). [^11^C]- MRB *BP*_ND_ for all subjects, in the selected ROIs, are reported in Supplementary Table S1. The regional average of the *BP*_ND_ images are highly correlated with the *BP*_ND_ values estimated from the analysis of the corresponding regional TACs, except in the locus ceruleus, where the smoothing applied to the dynamic images prior to parametric image computation introduces an underestimation of *BP*_ND_ values in this small ROI: regression line equations are *y* = 0.927 *x* + 0.026, *r* ^2^ = 0.964 for all ROIs except the locus ceruleus, with ROI TAC fit results on the x-axis and parametric images results on the y axis. Time activity curves for the thalamus and occipital cortex, at baseline and the 20mg dose, are shown in Supplementary Figure S1. Apparent NET occupancy, computed from *BP*_ND_ values and their standard errors from regional TAC fits, increased dose-dependently (Table 3). The relationship between estimated NET occupancy *r*_App_, estimated across several NET-rich brain regions and corrected for the maximal apparent occupancy (see Methods), and mean plasma concentration of TD-9855 during each post-dose PET scan is depicted in Figure 4. The best fit to the occupancy model indicated an EC_50_ of 1.21±0.21ng/mL.

**Table 3.** Apparent NET and SERT Occupancies (*r*_App_) Estimated from [^11^C]-MRB and [^11^C]-DASB Binding Potentials, Respectively.

**Subject**	TH-9855 Dose	NET Occupancy				SERT Occupancy
		Day 1	Day 3	Day 6	Day 7	Day 1
**1001**	20 mg	120.3±6.2			2.6±11.6	15.5±2.6
**1002**	20 mg	98.4±4.7		62.9±5.0		29.1±2.2
**1003**	20 mg	119.1±8.3	96.2±10.2			30.5±3.1
**1004**	20 mg	121.2±4.5	124.3±4.9			
**1006**	10 mg	110.3±7.4	92.0±10.8			
**1008**	4 mg	42.2±6.4	29.6±7.1			
**1010**	4 mg	57.9±4.7	57.3±3.8			
**1011**	4 mg	58.7±5.5	47.2±6.0		3.2±7.8	

Percent occupancy values were obtained from multiple regions and the weighted average of *r*_*App*_ and its associated standard error (see Methods) are reported in each case.

**Figure 5.**
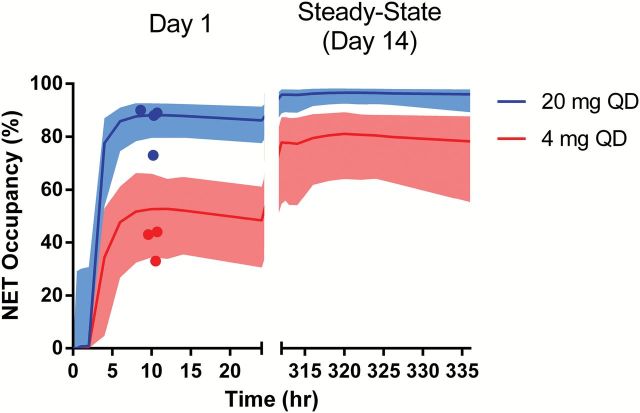
Simulation of NET Occupancy after single dose (Day 1) and repeated (steady state) administration of TD-9855 at 4mg and 20mg. Symbols represent observed NET occupancy determinations and solid lines indicate the mean simulated NET occupancy after TD-9855 administration on Day 1 and at steady state. Shaded bands indicate the 95% CI.

Simulation of the NET occupancy after repeated TD-9855 administration (4mg and 20mg) is presented in Figure 5. Repeat-dose simulations were based on a TD-9855 population PK model and the estimates of NET EC_50_ determined after single doses of TD-9855. Due to the long plasma t_1/2_of TD-9855 (30–40hr), accumulation of TD-9855 is observed over time in plasma and thus NET occupancy after repeated administration is higher than that observed after a single dose. Estimates of the NET maximal and trough mean occupancies at steady state indicate a greater than 75% occupancy for the 4mg dose. Both maximal and trough mean occupancies are predicted to exceed 95% at steady state for the 20mg dose. Corresponding data for all three dose levels (4, 10, and 20mg) are presented in Table 4. These estimates indicate that minimal (<10%) variation in peak and trough mean NET occupancy would be anticipated with repeated TD-9855 administration.

**Table 4.** Projected NET Occupancy at Steady State After TD-9855 Administration.

TH-9855 Dose (mg)	Projected Mean (Range) NET Occupancy	
	Max (Range)	Trough (Range)
20	**96** (91–98)	**95** (85–97)
10	**92** (84–96)	**91** (73–95)
4	**81** (64–89)	**77** (54–87)

Range reflects 95% CI.

**Figure 6.**
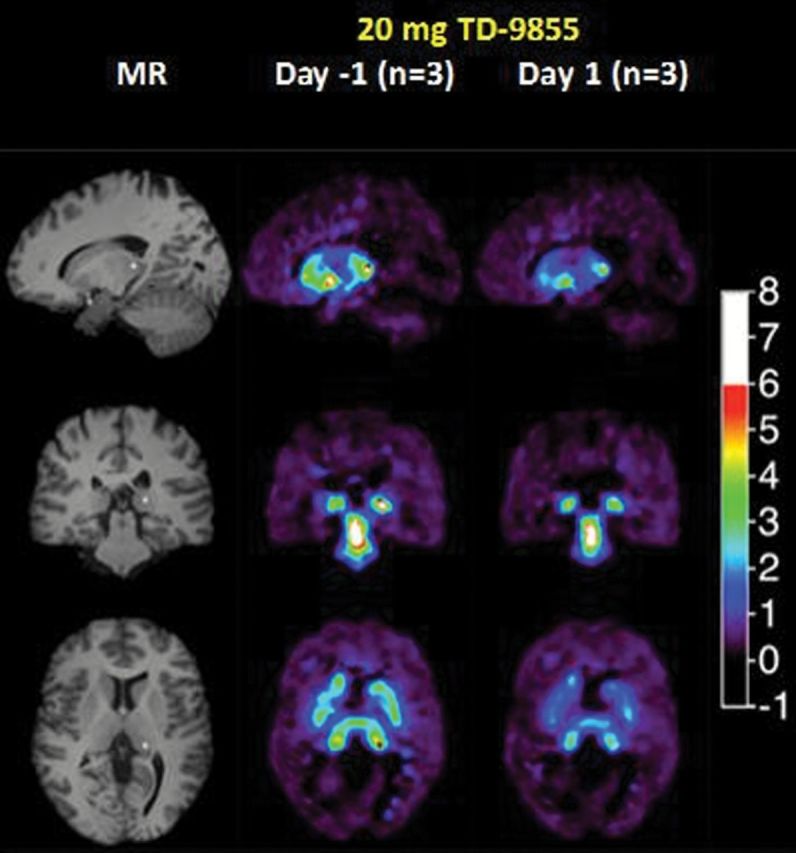
Average PET binding potential (*BP*_ND_) images of SERT with [^11^C]-DASB. Column 1 shows MR images in sagittal, coronal, and transverse orientation. Columns 2 and 3 show [^11^C]-DASB binding potential images at baseline and 7.5 to 9.5 hours after administration of 20mg of TD-9855. + shows the location of the orthogonal slices, cut through the thalamus.

Average SERT binding potential images for [^11^C]-DASB at baseline and at 7.5–9.5hr post-administration of 20mg of TD-9855 are shown in Figure 6. [^11^C]-DASB BP_ND_ in the selected ROIs are reported in Supplementary Table S2. The average SERT occupancy (mean ± standard deviation derived from individual values shown in Table 3), measured only on Day 1, was 25% ± 8% in the brain and corresponded to a mean plasma concentration of 6.35ng/mL (n = 3). Due to low levels of SERT occupancy (15–30%) at the single dose of 20mg and the limited number of available data points (n = 3), estimation of an EC_50_ value for SERT would be subject to significant uncertainty and estimation was not attempted.

Overall, TD-9855 treatment was well tolerated. No deaths, severe AEs, or AEs occurred during the study. No clinically significant abnormalities were noted with respect to the clinical laboratory values, physical examination findings, vital sign measurements, or ECG results.

## Discussion

This study describes the application of a preclinical to clinical translational strategy to the development of a novel CNS-penetrant NSRI, TD-9855. The results described establish the functional selectivity of TD-9855 for NET over SERT in both *in vitro* and *in vivo* preclinical models and confirm this profile using PET imaging of CNS transporter occupancy in humans. The sequential assessment of transporter occupancy in the same human subjects provided an explicit translation of the selectivity profile into the clinical setting. Collectively, these data establish a relationship between monoamine transporter occupancy and TD-9855 dose. Low doses of TD-9855 should preferentially engage NET, whereas higher doses can yield dual inhibition of NET and SERT. Differential target engagement can inform clinical evaluation in patients with CNS disorders that have distinct underlying mechanisms. To our knowledge a similar translational approach has not been used in the development of single or dual NE and/or 5-HT reuptake inhibitors, although such approaches have been reported in the discovery of other potential CNS therapeutics (Chang et al., 2011).

The modest functional selectivity of TD-9855 for human NE transporters *in vitro* differentiates it from many other reuptake inhibitors with NE transporter activity. For example, duloxetine and venlafaxine exhibit greater than 10-fold functional selectivity for 5-HT, whereas atomoxetine and esreboxetine exhibit high (≥20- and ≥10 000-fold, respectively) functional selectivity for NE (Wu et al., 2008; Tsuruda et al., 2010). TD-9855 exhibited high affinity binding to both human SERT and NET, although there was a small reduction in apparent NET selectivity between the functional and binding assays, as has been reported previously (Vaishnavi et al., 2004). Assay-dependent differences in transporter potency and binding affinity for monoamine reuptake inhibitors *in vitro* are frequently observed, although the mechanisms underlying these are unclear (Tsuruda et al., 2010).

TD-9855 demonstrated *in vivo* selectivity for NET in the *ex vivo* evaluation of transporter occupancy, consistent with the NET selectivity observed in the rat *in vitro* pharmacology studies. The estimates of the equilibration rate constant (k_eo_) were relatively large, indicating a rapid equilibration for TD-9855 within the CNS biophase (CNS equilibration t_1/2_ of ~0.4hr for NET and ~0.06hr for SERT). The transporter binding kinetics for TD-9855 are rapid at human NET and SERT *in vitro*. Association and dissociation rate constants at NET were 0.055nM^-1^min^-1^ and 0.069min^-1^, respectively, and were not determined at SERT as the binding kinetics were too rapid. These results are consistent with a profile of rapid brain entry and transporter binding kinetics for TD-9855 in rats. Therefore, the minor hysteresis suggested by the time course of NET occupancy versus TD-9855 plasma concentration (i.e., occupancy measured at the 0.5hr time point was slightly lower than that observed at similar plasma concentrations at ≥2hr post dose) cannot be explained by significantly-delayed entry of TD-9855 to the brain or slow transporter binding on/off rates (Yassen et al., 2005). It is possible that the observed hysteresis in rats reflects minor delays in the initial brain entry or the time required for distributional equilibrium within the CNS for TD-9855.

Similar to the rat PK/PD studies, the TD-9855 PET study was designed to estimate the CNS occupancy of NET and/or SERT at various time-points post dose. The advantage of this design over single-time point designs (e.g., scanning at the time of maximal plasma concentration) is the ability to evaluate the kinetics of occupancy over time, assess hysteresis in the relationship between occupancy and plasma concentration, and provide confidence in multiple-dose projections of occupancy from a single-dose PET study (Abanades et al., 2011). We observed no significant hysteresis in the relationship between NET occupancy and TD-9855 plasma concentration: i.e., the relationship established on Day 1 was similar to that observed after scanning at time points post-Day 1. Although it is possible that a larger dataset would have yielded the minor hysteresis effect we observed in rats, the pharmacokinetic profile is consistent with rapid penetration of TD-9855 into the human CNS, as would be anticipated for a non-P-gp substrate with high intrinsic permeability properties (Theravance Biopharma US, Inc., data on file).

In humans, the plasma EC_50_ for NET was slightly lower than that estimated from the translational rat PK/PD model (1.21ng/mL versus 5.5ng/mL), implying that NET occupancies were higher in humans than predicted from the preclinical model. Species differences in the *in vitro* binding to rat and human plasma proteins were taken into consideration when translating the rat PK/occupancy relationship to humans; however, these may not be representative of *in vivo* plasma/protein binding differences. No differences in transporter affinity were assumed during the translation, as NET binding affinity was similar between rat and human under comparable *in vitro* conditions. Furthermore, the functional inhibition profile of TD-9855 *in vitro* was not significantly different between species, and similar pharmacological inhibition would be anticipated in humans and rats *in vivo*.

A key limitation of the current study is that exploration of the SERT occupancy versus plasma concentration relationship was incomplete and an EC_50_ value could not be reliably determined. However, the sequential assessment of transporter occupancy in the same human subjects provided unequivocal evidence of selectivity for NET over SERT after a 20mg single dose. Preclinical PK/PD modeling predicts ~62% and ~26% occupancy at NET and SERT, respectively, following a single dose of 20mg. SERT occupancy following repeated administration of a 20mg dose in humans is predicted to be higher than that observed after a single dose, given the long plasma half-life (30–40hr) and approximately 3- to 4-fold accumulation. The preclinical PK/PD model predicts ~60% SERT occupancy at 20mg after repeated dosing. To determine the SERT EC_50_, a PET study incorporating either a higher single dose or repeated dose administration would be needed.

Mechanism-based PK/PD approaches, as used in the development of the current PK/PD models for TD-9855, have been applied in other transporter occupancy studies (Abanades et al., 2011; Bourdet et al., 2012). These approaches provide a description of the full time course of drug concentration in the plasma as it relates to the time course of changing transporter occupancy in the CNS. This allows for a more accurate projection of multiple-dose occupancy from single-dose preclinical and/or clinical PET studies. These approaches are particularly important for compounds that demonstrate some delay in distribution between the plasma and CNS or slow transporter binding kinetics. An important objective of the PET study was to support dose selection for further clinical development. Multiple factors must be taken into consideration when selecting doses for clinical evaluation, including the doses that will appropriately engage the target(s) as well as the mechanistic basis of the disorder of interest. Dual-monoamine reuptake inhibitors that target multidimensional CNS disorders amplify the challenge of dose selection, because differential target engagement may confer distinct benefits in different CNS disorders and/or comorbidities. Monoamine transporter occupancy studies have been used to understand the relationship between neurotransmitter systems in a disease state and the therapeutic efficacy observed in patients. For example, it is generally accepted that for clinical efficacy of SSRIs in MDD, SERT occupancy should exceed 80% (Meyer et al., 2001, 2004). In contrast, although explored in recent studies (Ding et al., 2014; Takano et al., 2014), the minimum level of NET occupancy required for clinical efficacy in disorders such as ADHD or MDD has not been established. In chronic pain inhibition of NET, but not SERT, alone is sufficient for analgesic efficacy (Rowbotham et al., 2004; Verdu et al., 2008; Hall et al., 2011; Arnold et al., 2012). However, the NET occupancy requirements and the contribution, if any, of SERT to the clinical efficacy of approved dual-reuptake inhibitors is unclear. At the approved dose (100mg) for management of fibromyalgia, milnacipran demonstrates ~40 % occupancy of both NET and SERT (Nogami et al., 2013). Recent imaging data (Sekine et al., 2010; Takano et al., 2014) also suggest that nortriptyline achieves ~50 % NET occupancy at a dose (75mg) that is similar to or greater than analgesic doses (Atkinson et al., 1998; Khoromi et al., 2007; Gilron et al., 2009). Duloxetine at the dose for chronic pain (60mg/day) achieves maximum SERT occupancy in the CNS (Takano et al., 2006; Abanades et al., 2011), but there are no corresponding data for NET.

The present data reveal a unique pharmacological profile of TD-9855 and describe doses that differentially engage NET alone or NET in conjunction with SERT. As the clinical profile of TD-9855 is established in patients with chronic pain or other CNS disorders, it may be possible to gain insights into the relative contributions of SERT and/or NET in these disorders.

In conclusion, using a translational approach we have established TD-9855 as a CNS-penetrant, NET-selective NSRI suitable for further investigation in patients with CNS disorders.

## Supplementary Material

For supplementary material accompanying this paper, visit http://www.ijnp.oxfordjournals.org/

## Statement of Interest

Funding for this study was provided by Theravance Biopharma US, Inc. Drs Smith, Bourdet, Daniels, Martin, Obedencio, Tsuruda, and Patil are current full-time employees and own equity securities of Theravance Biopharma US, Inc. Dr Kshirsagar was a paid consultant to Theravance Biopharma US, Inc. at the time the studies were conducted. Drs Kim, Daniels, Stangeland, and Patil were full-time employees of Theravance Biopharma US, Inc. at the time the studies were conducted.
